# Alcohol and Atrial Fibrillation

**DOI:** 10.31083/j.rcm2403073

**Published:** 2023-03-01

**Authors:** Stanisław Surma, Gregory Y.H. Lip

**Affiliations:** ^1^Faculty of Medical Sciences in Katowice, Medical University of Silesia, 40-752 Katowice, Poland; ^2^Liverpool Centre for Cardiovascular Science at University of Liverpool, Liverpool John Moores University and Liverpool Heart & Chest Hospital, L14 3PE Liverpool, UK; ^3^Department of Clinical Medicine, Aalborg University, 9220 Aalborg, Denmark

**Keywords:** atrial fibrillation, alcohol consumption, cardiovascular risk

## Abstract

Dietary habits, including alcohol consumption, are among the significant risk factors for the occurrence of atrial fibrillation (AF). The pathophysiological relationship between alcohol consumption and AF is complex and multifactorial. However, there is conflicting information about the impact of alcohol consumption (in various doses and types) on the risk of AF and AF-related outcomes. Alcohol consumption is significantly associated with AF in a gender-independent manner. The widespread belief that moderate amounts of alcohol, especially red wine, have cardioprotective effects may mean that more people will use alcohol. Even small amounts of alcohol regularly consumed increase the risk of AF. In this narrative review, we will review the epidemiological associations between alcohol and AF, and the implications for incident AF and AF-related outcomes.

## 1. Introduction

Atrial fibrillation (AF) is the most common cardiac arrhythmia in humans [1]. The 2019 global burden of AF is estimated at 59.7 million (95% confidence interval: 45.7 to 75.3 million), double the number of estimated cases in 1990 [2]. It is estimated that in 2050 the incidence of AF may increase by more than 60% [3]. Experts indicate that in 2050 the prevalence of AF in the USA and Europe will amount to 16 and 16–17 million cases, respectively [4]. The high prevalence of AF is a significant economic problem, associated primarily with frequent hospitalizations, absenteeism or complications of the disease, including stroke [5].

From a clinical point of view, an important issue is the effect of diet and lifestyle on the risk of AF [6]. Dietary habits, including alcohol consumption, are among the significant risk factors for the occurrence of AF. Manthey *et al*. [7] showed that the consumption of alcohol in the world is constantly increasing: 1990—5.9 L/*capita*; 2017—6.5 L/*capita*; 2030—7.6 L/person (in terms of pure ethanol). Alcohol use is a leading risk factor for global disease burden and causes substantial health loss [8]. According to World Health Organisation (WHO) worldwide, 3 million premature deaths every year result from harmful use of alcohol. This represents 5.3% of all deaths [9]. However, there is conflicting information about the impact of alcohol consumption (in various doses and types) on the risk of AF and AF-related outcomes.

In this narrative review, we will review the epidemiological associations between alcohol and AF, and the implications for incident AF and AF-related outcomes.

## 2. Alcohol Consumption—What the Guidelines and Recommendations Say

In scientific nomenclature, the basic way of determining the amount of alcohol consumed is the standard alcohol unit (SAU; drink), which is the amount of alcoholic beverage (of any kind) that contains 12.5 mL or 10 g of pure ethanol. For example: a small beer (330 mL; 4.5%) is 1.19 SAU; a large beer (500 mL; 4.5%) is 1.8 SAU; a glass of wine (175 mL; 12%) is 1.68 SAU; a shot of vodka (50 mL; 40%) is 1.6 SAU. There are other definitions of SAU. For example, in the UK it is the amount of an alcoholic drink that contains 10 mL or 8 g of pure ethanol. Another definition indicate that a unit of alcohol is 12 g of pure ethanol [10, 11].

In general, alcohol consumption has been defined as: light (<7 standard drinks/week); moderate (7 to 21 standard drinks/week); and heavy (>21 standard drinks/week), where 1 standard drink is approximately 12 g of alcohol [10, 11]. The guidelines of the European Society of Cardiology (ESC 2021) on cardiovascular prevention indicate that alcohol consumption should be limited to 100 g/week, both among women and men (recommendation class: 1; level of evidence: B) [12]. The Dietary Guidelines for Americans by Centers for Disease Control and Prevention recommend limiting alcohol consumption to 2 drinks or less in a day for men or 1 drink or less in a day for women, on days when alcohol is consumed [13].

## 3. Alcohol and Atrial Fibrillation

The association between excessive drinking and various forms of cardiovascular disease is well established [14]. The influence of the amount of alcohol consumed on the risk of most cardiovascular diseases takes the shape of a “J” curve [14]. Considering the constantly increasing prevalence of AF, it is clinically important how the relationship between the amount of alcohol consumed and the risk of this arrhythmia develops.

### 3.1 A Brief Overview of Pathophysiological Mechanisms

Alcohol may act proarrhythmogenic in many mechanisms. At the cellular level, alcohol can damage intercellular junctions and cells, trigger inflammation and oxidative stress, and disrupt the regulation of ion channels in the myocardium [14, 15]. Within the autonomic nervous system, alcohol increases the activity of the sympathetic component and reduces heart rate variability (HRV) [14, 15].

Moreover, alcohol promotes dilatation and fibrosis of the left atrium and increases its pressure [16, 17]. Long-term alcohol consumption promotes atrial cardiomyopathy (alcoholic cardiomyopathy) associated with structural, functional and electrical remodeling of the atria, thus stabilizing AF episodes and contributing to AF progression [16, 17]. In addition, alcohol consumption often leads to other risk factors for AF, such as being overweight, obstructive sleep apnea, and arterial hypertension [16]. Myocardial hypertrophy resulting from chronic alcohol consumption by increasing the reactivity of transient receptor potential cation channels (TRPC) leads to electrical instability of cardiomyocytes, which directly contributes to the occurrence of arrhythmias [18, 19].

Alcohol directly causes a number of disorders in the electrophysiology of the heart. This effect is related to the dysregulation of ion channels. One *in silico* study found that alcohol reduced $I_{\text{Na}}$, $I_{\text{Ca,L}}$, $I_{\text{to}}$, $I_{\text{Kr}}$ and $I_{\text{Kur}}$, dual effects on $I_{\text{K1}}$ and $I_{\text{K,ACh}}$ (inhibition at low and augmentation at high concentrations), and increased $I_{\text{NCX}}$ and sarcoplasmatic reticulum (SR) ${\mathrm{Ca}}^{2+}$ leak and SERCA uptake in cardiomyocytes. Alcohol may also damage the gap junctions between cardiomyocytes [20]. Finally, alcohol consumption leads to decreased biatrial conduction velocities (CV), action potential duration and refractoriness, and shortened right atrial effective refractory periods (AERP) while increasing the dispersion of atrial refractoriness [20].

Heavy alcohol consumption can result in the holiday heart syndrome, which was first described in 1978 and occurs when healthy subjects without heart disease known to cause arrhythmia experience AF after excessive alcohol consumption, which was observed mainly after weekends or holidays [21]. This can probably be attributed to such effects of alcohol as: inhibition of sodium channels in myocardial cells, stimulation of the sympathetic and parasympathetic nervous system, increase in the concentration of free fatty acids in the blood and stimulation of c-Jun N-terminal kinase 2 (JNK2) kinase [secondarily leads to the stimulation of calmodulin II kinase (CaMKII) and the release of calcium ions from the SR, which ultimately contributes to the development of AF], and above all - electrolyte changes, including hypokalemia and hypomagnesemia, after taking large doses of alcohol in a short time [21, 22, 23].

**Fig. 1.**
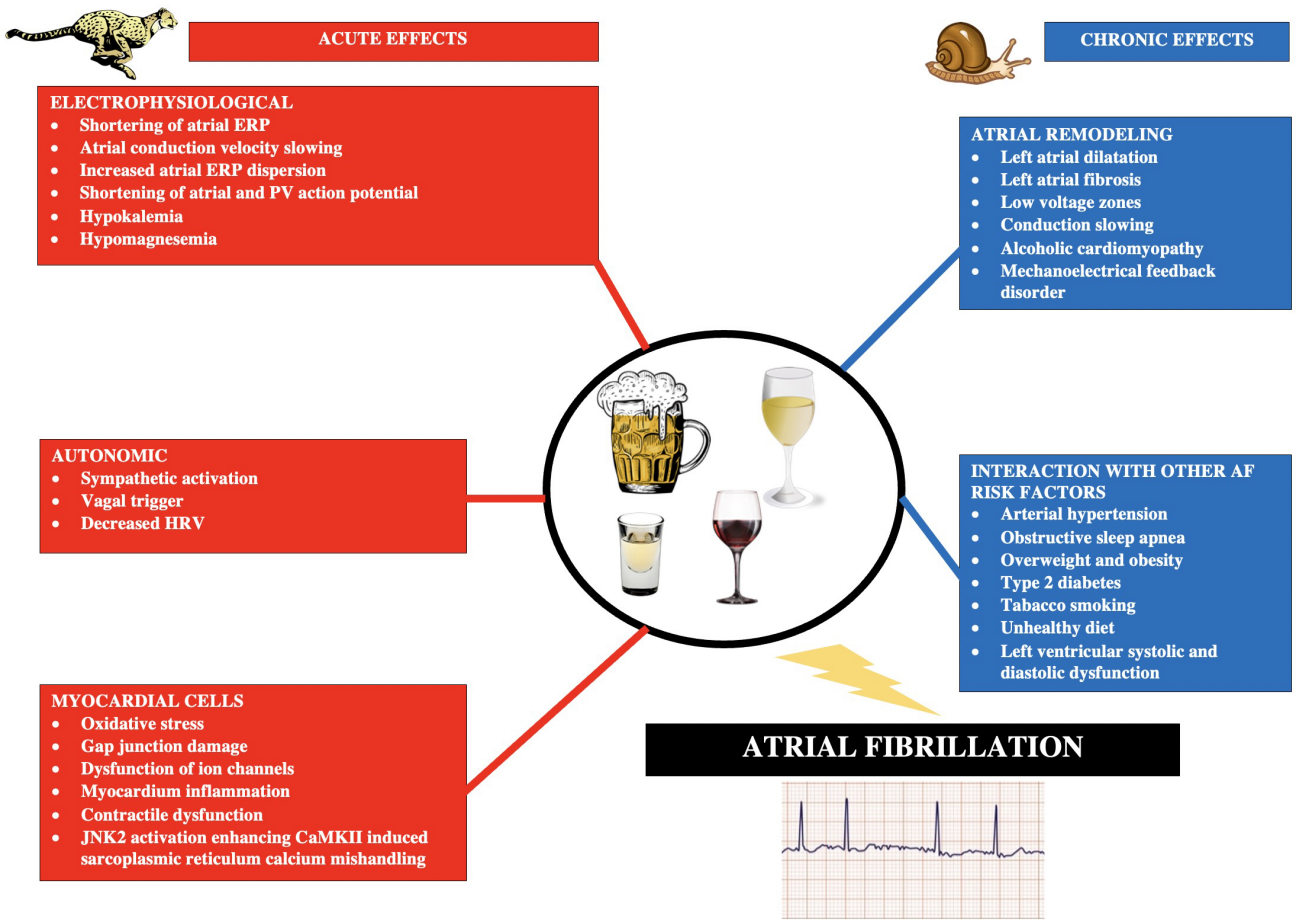
**Main acute and chronic pathophysiological mechanisms triggered by alcohol consumption and leading to the development of atrial fibrillation**. Based on [14, 15, 27, 28]. Abbreviations: AF, atrial fibrillation; ERP, effective refractory period; PV, pulmonary vein; HRV, heart rate variability; JNK2, c-Jun N-terminal kinase 2; CaMKII, calmodulin II kinase.

In a study by Voskoboinik *et al*. [24] including 75 patients undergoing AF ablation using high density electroanatomical mapping, moderate alcohol consumption (8–21 drinks/week) was found to be associated with lower bipolar voltage and slower atrial conduction velocity. In a randomized and placebo-controlled study by Marcus *et al*. [25], covering 100 subjects, acute exposure to alcohol reduces atrial effective refractory periods (AERP), particularly in the pulmonary veins, which is substrate for AF. In a study by Sha *et al*. [26], including 134 patients with AF, alcohol causes echocardiographic and electrophysiological changes such as impaired peak left atrial longitudinal strain, obvious inter-atrial conduction delay and increasing ERP dispersion. Intra-atrial conduction delay and ERP dispersion also increased with increasing levels of alcohol consumption [26]. The most important mechanisms of the influence of alcohol consumption on the pathogenesis of AF are summarized in Fig. 1 (Ref. [14, 15, 27, 28]).

The safer effect of drinking wine, observed in some studies [17], in contrast to other alcohols, may be due to the richness of polyphenols contained in this red and white beverage. Of particular interest is resveratrol, a polyphenol found in large amounts in red wine [29]. Resveratrol has been shown to reduce the inducibility of AF, potentially by reducing left atrial fibrosis and regulating ion channel function [30]. The overall effect of polyphenols on reducing the risk of AF is associated with, among others, anti-inflammatory, antioxidant and anti-fibrotic effects, as is the case with the consumption of polyphenol-rich coffee [31].

In summary, alcohol consumption triggers both acute and chronic mechanisms that lead to the onset of AF. Regular consumption of even ESC-acceptable alcohol doses (100 g/week) leads to electrical and structural remodeling of the atria and increases the risk of AF. The polyphenols contained in wine can to some extent mitigate the negative effects of alcohol itself.

### 3.2 Dose of Alcohol

The most important results relating alcohol consumption and AF are summarized in Table 1 (Ref. [17, 32, 33, 34, 35, 36, 37, 38, 39, 40, 41, 42, 43, 44, 45, 46, 47, 48]).

**Table 1.** **Summary of the results of the most important studies and meta-analyses evaluating the impact of alcohol consumption on the risk of developing atrial fibrillation**.

Author, year, [Ref]	Meta-analysis/study characteristics	The amount of alcohol consumed and the risk of AF		Notes/Comment	What pattern of alcohol consumption increases the risk of AF?
				Compatibility with the ESC 2021 guidelines	
Samokhvalov A. *et al*., 2010, [32]	6 studies/67,891 subjects	12 g/day	F and M: RR = 1.08; 95% CI: 1.02–1.14	Gender - did not affect the risk of AF Dose - The greater the alcohol consumption, the greater the risk of AF ESC 2021: no	∙ Any amount of alcohol (small consumption - small effect)
		24 g/day	F: RR = 1.07; 95% CI: 1.04–1.10		
			M: RR = 1.08; 95% CI: 1.04–1.11		
		60 g/day	F: RR = 1.42; 95% CI: 1.23–1.64		
			M: RR = 1.44; 95% CI: 1.23–1.69		
		120 g/day	F: RR = 2.02; 95% CI: 1.60–2.97		
			M: RR = 2.09; 95% CI: 1.52–2.86		
Kodama S. *et al*., 2011, [33]	14 studies/138,378 subjects	High *versus* low	RR = 1.51; 95% CI: 1.31–1.74	Gender, comorbidities and reference group (low or no intake) did not influence the risk of AF ESC 2021: yes	∙ Increased alcohol consumption
Gallagher C. *et al*., 2017, [34]	9 studies/249,496 subjects	Low (1 drink/day)	HR = 0.95; 95% CI: 0.85–1.06	Moderate alcohol consumption significantly increased the risk of AF only in men ESC 2021: yes	∙ High consumption in both sexes ∙ Moderate consumption in men
		Moderate	HR = 1.11; 95% CI: 1.05–1.18		
		High *versus* low/no consumption	HR = 1.34; 95% CI: 1.20–1.49		
Larsson S. *et al*., 2014, [35]	7 prospective studies/205,073 subjects	Increase alcohol intake by an additional drink (12 g pure alcohol)/day	RR = 1.08; 95% CI: 1.06–1.10	The risk was not affected by: gender, geographical origin	∙ Each increase in alcohol consumption by one drink/day
				ESC 2021: no	
Yang L. *et al*., 2022, [36]	13 studies/10,266,315 subjects	Small (about 1 drink/day)	HR = 1.05; 95% CI: 0.98–1.13	Small F and M - no effect Moderate F - no effect M - (HR = 1.09; 95% CI: 1.07–1.11) Region: Europe Small - HR = 1.09; 95% CI: 1.02–1.17 Moderate - HR = 1.26; 95% CI: 1.09–1.44 Types of alcohol (spirits, beer, wine) Small - no impact Moderate - no impact ESC 2021: yes	∙ Moderate consumption in men ∙ Small and moderate among Europeans
		Moderate (about 2 drinks/day)	HR = 1.14; 95% CI: 1.07–1.21		
Zhang H. *et al*., 2022, [37]	13 prospective studies/668,905 subjects	Small (<12 g/day)	F: HR = 0.97; 95% CI: 0.90–1.04	Gender had some effect on the risk of AF associated with alcohol consumption ESC 2021: yes	∙ Moderate to high in men ∙ Large in women
			M: HR = 1.04; 95% CI: 0.97–1.11		
		Moderate (12–24 g/day)	F: HR = 1.02; 95% CI: 0.91–1.14		
			M: HR = 1.21; 95% CI: 1.10–1.33		
		High (>24 g/day)	F: HR = 1.32; 95% CI: 1.10–1.60		
			M: HR = 1.54; 95% CI: 1.26–1.89		
Giannopoulos G. *et al*., 2022, [38]	16 studies/13,044,007 subjects	Moderate (<168 g/week)	logOR = –0.20; 95% CI: –0.28 to –0.12	The dependence taking the shape of the “J” curve was found ESC 2021: yes	∙ More than 14 drinks/week
		High (>168 g/week)	logOR = 0.14; 95% CI: 0.01–0.2		
Jiang H. *et al*., 2022, [39]	13 prospective studies/10,151,366 subjects	Each increase in alcohol consumption by one drink	F: RR = 1.05; 95% CI: 0.96–1.14	Women: J-curve association Men: linear association ESC 2021: no	∙ Any amount in men ∙≥3.5 drinks/day for women
			M: RR = 1.08; 95% CI: 1.05–1.11		
Ariansen I. *et al*., 2012, [40]	9193 patients with LVH and AH	>10 drink/week	HR = 1.60; 95% CI: 1.02–2.51	Multivariate analysis ESC 2021: yes	∙>10 drink/week
		<10 drink/week	Statistically insignificant risk		
Larsson S. *et al*., 2014, [35]	Prospective/79,019 subjects	1–6 drinks/week	RR = 1.01; 95% CI: 0.94–1.09	Comparison: <1 drink/week Multivariate analysis: age, gender, weight, smoking, comorbidities Type of alcohol and a significant increase in the risk of AF: Liqueur: from 7 drinks/week Wine: from 14 drinks/week Beer: inconsistent data ESC 2021: yes	∙>15 drinks/week
		7–14 drinks/week	RR = 1.07; 95% CI: 0.98–1.17		
		15–21 drinks/week	RR = 1.14; 95% CI: 1.01–1.28		
		>21 drinks/week	RR = 1.39; 95% CI: 1.22–1.58		
Cha M. *et al*., 2020, [41]	Prospective/19,634 subjects	Drinking *versus* not drinking	HR = 2.21; 95% CI: 1.55–3.14	Men more predisposed to alcohol-related AF ESC 2021: no	∙ Drinking alcohol, especially in larger amounts
		Higher *versus* lower consumption	HR = 3.15; 95% CI: 1.98–4.99		
Csengeri D. *et al*., 2021, [42]	Prospective/107,845 subjects	1 g/day (0.08 drink)	HR = 1.01; 95% CI: 0.99–1.04	No significant differences between vodka and beer. The risk of AF associated with wine consumption was the least expressed or insignificant ESC 2021: no	∙ Regular consumption of even small amounts of alcohol, especially spirits and beer
		2 g/day (0.17 drink)	HR = 1.02; 95% CI: 1.0–1.04		
		3 g/day (0.25 drink)	HR = 1.04; 95% CI: 1.02–1.05		
		4 g/day (0.33 drink)	HR = 1.05; 95% CI: 1.03–1.07		
		5 g/day (0.42 drink)	HR = 1.06; 95% CI: 1.04–1.08		
		6 g/day (0.5 drink)	HR = 1.07; 95% CI: 1.05–1.1		
		12 g/day (1 drink)	HR = 1.16; 95% CI: 1.11–1.22		
		24 g/day (2 drinks)	HR = 1.36; 95% CI: 1.25–1.47		
		36 g/day (3 drinks)	HR = 1.52; 95% CI: 1.35–1.7		
		48 g/day (4 drinks)	HR = 1.59; 95% CI: 1.37–1.85		
		60 g/day (≥5 drinks)	HR = 1.61; 95% CI: 1.35–1.92		
Tu S. *et al*., 2021, [17]	Prospective/403,281 subjects	Increasing doses of alcohol	J-shaped relationship in total alcohol consumption, with the lowest risk of AF at less than 7 drinks/week	The strength of the study: comparable numbers of women and men.	∙ Beer and cider - any amount
				Beer and cider - any consumption is harmful	∙ Red wine >10 drinks/week
				Red and white wine and spirits: up to 10, 8 and 3 drinks/week, respectively, not associated with an increased risk of AF	∙ White wine >8 drinks/week
				Gender - no significant differences were found	∙ Spirits >3 drinks/week
				ESC 2021: no	
Han M. *et al*., 2022, [43]	Prospective/1,537,836 subjects	105–210 g/week	HR = 1.25; 95% CI: 1.12–1.40	Multivariate analysis - no effect on AF risk ESC 2021: yes	∙ Moderate ∙ High
		≥210 g/week	HR = 1.47; 95% CI: 1.18–1.83		
Marcus G. *et al*., 2022, [44]	RCT, n-of-1 trial/446 patients	Acute exposure to alcohol	OR = 2.15; 95% CI: 1.17–3.61	ESC 2021: no	Acute exposure to alcohol
Marcus G. *et al*., 2021, [45]	100 subjects/Real-time detection of AF	1 drink	After 4 hours: OR = 2.02; 95% CI: 1.38–3.17	Individual AF episodes were associated with higher odds of recent alcohol consumption ESC 2021: no	∙ Even 1 drink
		2 drinks	After 4 hours: OR = 3.58; 95% CI: 1.63–7.89		
Aung S. *et al*., 2022, [46]	36,158 subjects	Occasional increase in alcohol consumption (weekend, holiday, etc.)	Statistically significant increase in ED visits for AF and for new-onset AF (1757 additional visits for new-onset, incident AF ED visits/100,000 person-years; 95% CI: 945–2.569 visits, *p *< 0.001) during and shortly after periods of increased alcohol consumption	Population-based study	∙ Occasional increase in alcohol consumption
				ESC 2021: no	
Frederiksen T.C. *et al*., 2022, [47]	43,758 subjects/Five-year changes in alcohol intake	≤6.9 drinks/week	Females: ≤6.9 to ≥21 drinks/week → increase risk by 45% (HR = 1.45; 95% CI: 1.07–1.98); 7–13.9 to ≥21 drinks/week → increase risk by 30% (HR = 1.30; 95% CI: 1.04–1.64). Males: increasing alcohol consumption did not affect the risk of AF	Multivariate analysis There were no differences in the effect of different types of alcohol on the risk of AF ESC 2021: yes	∙ Increase in alcohol intake was associated with a greater risk of AF compared with a stable low/moderate intake
		7–13.9 drinks/week			
		14–20.9 drinks/week			
		≥20 drinks/week			
Biddinger K.J., 2022, [48]	371,463 subjects from UK Biobank	Consumption *versus* non-consumption (Mendelian randomization)	OR = 1.24; 95% CI: 1.08–1.44	Causality assessment Analysis with the exclusion of abstainers ESC 2021: no	∙ Any amount of alcohol
		Non-consumption and 7, 14, 21 and 28 drinks/week	The relationship between alcohol and the risk of AF was dose-dependent. Already from the smallest dose of alcohol (1 drink/week) the risk of AF began to increase statistically significantly		

Abbreviations: AF, atrial fibrillation; ESC, European Society of Cardiology; F, female; M, male; RR, relative risk; 95% CI, 95% confident interval; HR, hazard ratio; OR, odds ratio; LVH, left ventricular hypertrophy; AH, arterial hypertension; RCT, randomized controlled trial; ED, emergency department.

In a meta-analysis of 6 studies the risk of AF was significantly increased from the consumption of 12 g or more of alcohol/day. A linear dose-response relationship has been demonstrated between alcohol consumption and the risk of AF [32]. A meta-analysis of 14 studies found that higher (*versus* lower) alcohol consumption was associated with a significant increase in the risk of AF (RR = 1.51; 95% CI: 1.31–1.74). A linear dose-response relationship has been demonstrated between alcohol consumption and the risk of AF [33]. A meta-analysis of 9 studies (249,496 subjects) found that heavy to moderate alcohol consumption was significantly associated with an increased risk of AF (by 34% and 11%, respectively). Low alcohol consumption was not associated with the risk of AF [34]. In a meta-analysis of 7 prospective studies (205,073 participants), it was found that each one drink/day increase in alcohol consumption was significantly associated with an 8% increase in the risk of AF (RR = 1.08; 95% CI: 1.06–1.10) [35]. One meta-analysis of 13 studies (10,266,315 subjects) found that regular moderate (about 2 units of alcohol/day) alcohol consumption increased the risk of AF (HR = 1.14; 95% CI: 1.07–1.21), while low alcohol consumption (about 1 unit of alcohol/day) had no effect on this risk [36]. Another meta-analysis of 13 studies (668,905 subjects) found that heavy alcohol consumption increased the risk of AF by 30% (HR = 1.30; 95% CI: 1.20–1.41), moderate by 12% (HR = 1.12; 95% CI: 1.06–1.18), while low did not affect the risk (HR = 1.00; 95% CI: 0.96–1.05) [37].

Nonetheless, a meta-analysis of 16 studies (13,044,007 subjects) showed a slightly different relationship between alcohol consumption and the risk of AF. A relationship in the form of a “J” curve was found, as high alcohol consumption (>168 g/week) was significantly associated with an increased risk of AF, while moderate consumption (<168 g/week) was characterized by a protective effect [38]. However, the results of this meta-analysis were not confirmed in meta-analysis of 13 prospective studies, covering over 10 million subjects, conducted by Jiang *et al*. [39]. This meta-analysis found that each increase in alcohol consumption by one drink/day is significantly associated with an increased risk of AF (RR = 1.06; 95% CI: 1.03–1.08) [39].

**Fig. 2.**
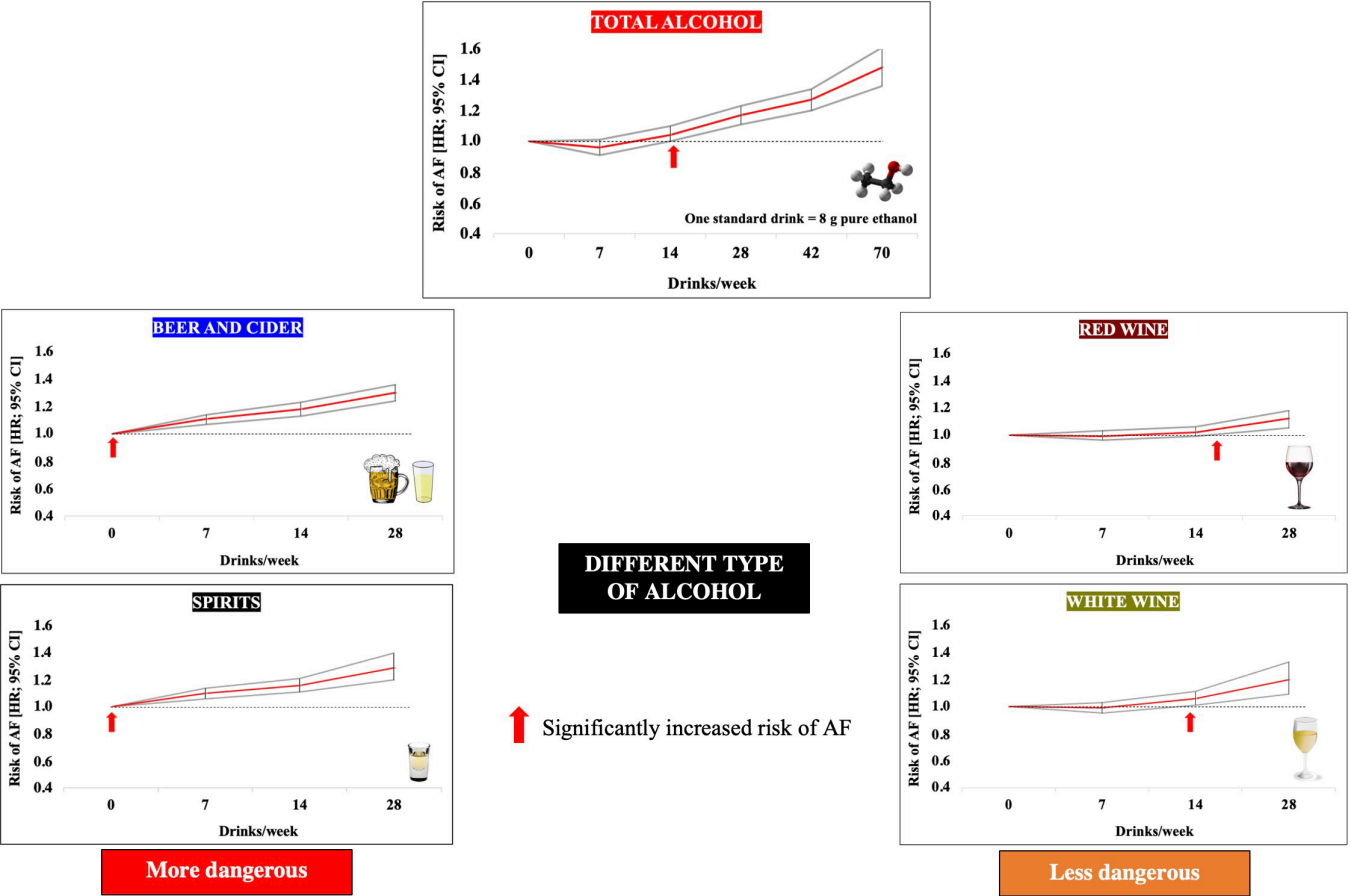
**Effect of alcohol consumption and different types of alcoholic beverages on the risk of developing atrial fibrillation**. Data adapted from [17]. Abbreviations: AF, atrial fibrillation; HR, hazard ratio.

In the losartan intervention for endpoint Reduction in Hypertension (LIFE) randomized clinical trial, including 9193 patients with electrocardiography (ECG) signs of left ventricular hypertrophy and arterial hypertension, the consumption of >10 units of alcohol/week increased the risk of AF by 60% (HR = 1.60; 95% CI: 1.02–2.51) [40]. In a prospective study including 79,019 subjects who were followed for 11 years, the consumption of 1–6 alcohol units/week was not associated with the risk of AF (RR = 1.06; 95% CI: 0.98–1.15). Consumption of 7–14, 15–21 and >21 alcohol units/week significantly increased the risk of AF by 12%, 18% and 43%, respectively [35]. In a study by Cha *et al*. [41], including 19,634 subjects who were followed for 7.0 ± 2.8 years, the risk of AF was significantly higher in alcohol drinkers *versus* nondrinkers (HR = 2.21; 95% CI: 1.55–3.14) and higher in subjects drinking more *versus* less alcohol (HR = 3.15; 95% CI: 1.98–4.99). In a cohort study by Csengeri *et al*. [42] including 107,845 subjects from Europe, the effect of the amount of alcohol consumed on the risk of AF was analyzed during a 13.9-year follow-up, whereby regular consumption of one drink/day was associated with a 16% increased risk of AF (HR = 1.16; 95% CI: 1.11–1.22). Indeed, a statistically significantly increased risk of AF was found with regular alcohol consumption at the level of 2 g/day [42]. From a methodological point of view, the most convincing results come from a prospective study by Tu *et al*. [17], involving 403,281 subjects from UK Biobank. In this study, the number of women and men was comparable, and the follow-up period was 11.4 years. A J-shaped relationship was found between the amount of alcohol consumed and the risk of AF. The lowest risk of AF was observed with less than 7 drinks/week (56 g/week). Above this amount, the risk of AF started to increase (significant increase risk of AF: >14 standard drink/week; 112 g pure ethanol/week) (Fig. 2, Ref. [17]). In a cohort study by Han *et al*. [43] including 1,537,836 adults aged 20 to 39 years, it was found that regular moderate (105–210 g/week) to heavy (≥210 g/week) alcohol consumption significantly increases the risk of AF by 25% and 47%, respectively (HR = 1.25; 95% CI: 1.12–1.40 and HR = 1.47; 95% CI: 1.18–1.83).

In an observational study by Frederiksen *et al*. [47] of 43,758 participants without AF at baseline, increasing alcohol consumption over a 5-year follow-up was associated with a higher risk of AF [from ≤6.9 drinks/week to ≥21 drinks/week (HR = 1.38; 95% CI: 1.09–1.72) or from 14–20.9 drinks/week to ≥21 drinks/week (HR = 1.27; 95% CI: 1.01–1.59)]. Biddinger *et al*. [48] reported an analysis of 371,463 subjects from UK Biobank, where any amount of alcohol (from 1 drink/week) was significantly associated with an increased risk of AF.

In the I-STOP-AFib randomized clinical trial of 466 patients, acute exposure to alcohol increased AF risk (OR = 2.15; 95% CI: 1.17–3.61), with no evidence that other exposures, including caffeine, triggered AF [44]. In another study by Marcus *et al*. [45], including 100 subjects, 56 of whom had at least 1 episode of AF, within 4 hours of consuming one drink, the risk of AF (real-time documentation of each alcoholic drink consumed was self-recorded using a button on the ECG recording device) was doubled (OR = 2.02; 95% CI: 1.38–3.17), with a greater than 3-fold higher odds of at least 2 drinks (OR = 3.58; 95% CI: 1.63–7.89).

In a study covering 36,158 individuals, occasional (holiday, etc.) increases in alcohol consumption were associated with a statistically significant increase in ED visits for AF and for new-onset AF, with 1757 additional visits for new-onset, incident AF ED visits/100,000 person-years (95% CI: 945–2569 visits, *p *< 0.001) during and shortly after periods of increased alcohol consumption [46]. The increased occurrence of AF after heavy alcohol consumption during weekend or holidays is known as holiday heart syndrome (HHS) [49].

In summary, most studies provide evidence of a strong association between alcohol consumption and the risk of AF. It cannot be arbitrarily concluded that light or moderate alcohol consumption (up to 100 g/week) does not affect the risk of AF, as even small amounts of alcohol regularly consumed increase the risk of AF.

### 3.3 Type of Alcohol

A prospective study by Larsson *et al*. [35] found that for liquor, the risk of AF was increased when drinking 7–14 and >14 units of alcohol/week; while in the case of wine, consumption >14 alcohol units/week was associated with an increased risk of AF, and for beer consumption, 7–14 alcohol units/week. No significant differences in the effects of spirits or beer on AF risk were found in a prospective study by Csengeri *et al*. [42], where the risk of AF associated with wine consumption was the least pronounced or insignificant. In the meta-analysis by Yang *et al*. [36], analyzing the same types of alcohol, no significant differences in their impact on the risk of AF were found. In a prospective study by Tu *et al*. [17], beverage-specific analyzes have shown detrimental associations between any beer/cider consumption and the risk of AF, while for consumption of red and white wine and spirits up to 10, 8 and 3 drinks/week, respectively, no harmful effects were observed (Fig. 2). In an observational study by Frederiksen *et al*. [47] of 43,758 participants without AF at baseline no differences in the effect of different types of alcohol (beer, wine, spirit or liqueur) on the risk of AF were found.

In summary, the risk of AF depends on the type of alcohol consumed (highest: beer, cider and spirits; lowest: white and red wine).

### 3.4 Sex

Women consume significantly less alcohol compared to men. In a meta-analysis by Kodama *et al*. [33], heavy alcohol consumption increased the risk of AF in both women and men (37% and 32%, respectively). A similar finding was made in the meta-analysis by Samokhvalov *et al*. [32] showing an increased risk of AF in both women and men with 12 g of alcohol/day and more). In a prospective study by Tu *et al*. [17] involving an equal number of women and men (52.4% *versus* 47.6%), no significant sex differences in the risk of AF associated with alcohol consumption were found.

A meta-analysis by Gallagher *et al*. [34] found that moderate alcohol consumption significantly increased the risk of AF in men (HR = 1.26; 95% CI: 1.04–1.54) but not in women (HR = 1.03; 95% CI: 0.85–1.24). In a study by Cha *et al*. [41], men were also more predisposed to a higher risk of AF related to alcohol consumption compared to women. A greater susceptibility of men to develop AF in relation to alcohol was also found in the meta-analysis by Yang *et al*. [36] who found that although low alcohol consumption did not affect the risk of AF, moderate alcohol consumption increased it among men (HR = 1.09; 95% CI: 1.07–1.11) but not among women. A meta-analysis by Zhang *et al*. [37] found that moderate and heavy alcohol consumption was significantly associated with the risk of AF among men (HR = 1.21; 95% CI: 1.10–1.33 and HR = 1, respectively, 54; 95% CI: 1.26–1.89), while among women this was only evident with high alcohol consumption (HR = 1.32; 95% CI: 1.10–1.60). In a meta-analysis by Jiang *et al*. [39], the relationship between alcohol consumption and the risk of AF is linear among men, while among women it takes the shape of a “J” curve. In an observational study by Frederiksen *et al*. [47] of 43,758 participants without AF at baseline was found that increasing alcohol consumption in a 5-year follow-up significantly increased the risk of AF only among women.

Some [29, 31, 32, 34, 36], but not all [15, 27, 28, 47], studies have found a higher risk of AF in men who consume alcohol. Several studies have failed to show an increase in the risk of AF at all levels of alcohol consumption in women. Most of these earlier studies included only a few women who consumed large amounts of alcohol.

In summary, sex does not seem to have a significant effect on the risk of AF associated with alcohol consumption.

### 3.5 Genetic Predisposition

An important issue is whether certain gene polymorphisms of enzymes involved in alcohol metabolism (alcohol dehydrogenase—ADH; aldehyde dehydrogenase—ALDH) [50] may affect the risk of AF associated with alcohol consumption.

In a study by Tolstrup *et al*. [51], covering 88,782 subjects, *ADH1B/ADH1C* genetic variants were not found to affect the risk of AF associated with alcohol consumption. In a Mendelian randomized study (approximating the possibility of causal inference) by Yang *et al*. [52] including 8964 subjects, the rs671 polymorphism of the *ALDH2* gene (decreased activity resulting in greater accumulation of the highly toxic acetaldehyde) showed a significant association with the risk of AF in men (OR = 1.65; 95% CI: 1.06–2.67). In Mendelian randomization, genetically predicted daily alcohol consumption was positively associated with the risk of AF in both sexes (OR = 3.17; 95% CI: 1.18–9.24) [52]. In a study by Yamashita *et al*. [53], including 656 subjects, the ${\mathrm{ALDH2}}^{*}$${\mathrm{1/}}^{*}$2 carriers who habitually consume alcohol had a higher risk of AF (OR = 5.07; 95% CI: 2.03–12.70), which was associated with a lower rate of alcohol metabolism.

In a study by Biddinger *et al*. [48] of 371,463 participants from UK Biobank, which used Mendelian randomization (promoting the possibility of causal inference), alcohol consumption was significantly associated with the risk of AF (OR = 1.24; 95% CI: 1.08–1.44).

In summary, genetic polymorphisms of the genes of enzymes involved in ethanol metabolism may modulate the risk of AF associated with alcohol consumption.

## 4. Reduction of Alcohol Consumption and Risk of AF

### 4.1 Subjects without AF at Baseline 

In the Atherosclerosis Risk in Communities (ARIC) Study 15,222 subjects followed for 19.7 years, current and former drinkers were found to be at higher risk of AF than those who never drank alcohol, and every decade abstinent from alcohol was associated with an approximate 20% (95% CI: 11–28%) lower rate of incident AF [54]. Conversely, every additional decade of past alcohol drinking was associated with a 13% (95% CI: 3–25%) higher rate of AF and every additional drink/day during former drinking was associated with a 4% (95% CI: 0–8%) higher rate of AF [54]. The results of this study indicate that the lowest risk of AF was among subjects who had never consumed alcohol. In a study by Choi *et al*. [55], covering 1,112,682 patients with type 2 diabetes who were followed for 4 years, alcohol abstinence was associated with a low risk of AF development (HR = 0.81; 95% CI: 0.68–0.97).

In summary, reducing alcohol consumption is associated with a significant decrease risk of incident AF.

### 4.2 Patients with AF

In a meta-analysis of 9 studies (5436 patients) conducted by Grindal *et al*. [56], including patients after catheter ablation due to AF, the impact of alcohol consumption on the risk of recurrence of arrhythmia showed that compared with patients who consumed little or no alcohol, those who consumed moderate or high amounts of alcohol had a higher probability of recurring AF (OR = 1.45; 95% CI: 1.06–1.99).

In a randomized clinical trial including 140 patients with AF, the effect of reducing the amount of alcohol consumed (from 16.8 ± 7.7 to 2.1 ± 3.7 standard drinks/week; reduction by 87.5%) on the incidence of AF recurrence, with a 45% reduction in the risk of AF recurrence (HR = 0.55; 95% CI: 0.36–0.84), which was maintained for 6 months of follow-up [57]. In a prospective study by Takahashi *et al*. [58], including 3474 patients undergoing catheter ablation due to AF, reducing alcohol consumption (from 140 g/week to approximately 70 g/week) significantly reduced the risk of AF recurrence or atrial tachycardia by 37% (HR = 0.63; 95% CI: 0.52–0.77), compared to a lower reduction of alcohol consumption. In a similar study by Sagawa *et al*. [59] on 110 patients with AF without structural heart disease who underwent single ablation, the success rate after a single ablation was significantly lower in drinkers than in abstainers (79.3% *versus* 95.9% at 12 months; mean follow-up, 18 ± 8 months, *p* = 0.013).

In summary, reducing alcohol consumption significantly reduces the risk of AF recurrence in patients after catheter ablation.

## 5. Summary and Practical Conclusions

**Fig. 3.**
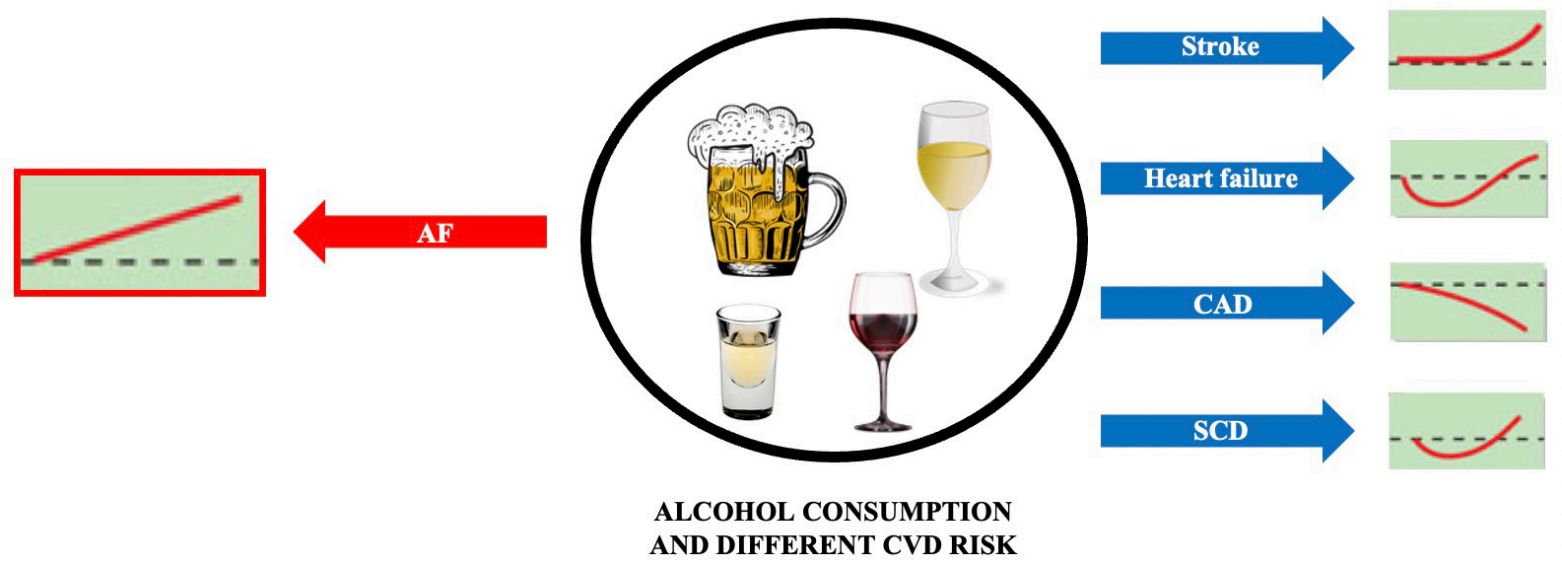
**Alcohol consumption and the risk of different cardiovascular diseases**. Based on [60]. Abbreviations: AF, atrial fibrillation; CAD, coronary artery disease; SCD, sudden cardiac death.

The effect of alcohol consumption on the risk of AF is different from other cardiovascular diseases (Fig. 3, Ref. [60]). Based on current knowledge, it is not possible to establish a completely safe amount of alcohol that would not increase the risk of AF. In patients with new onset AF, limiting alcohol consumption should be recommended, or be advised to change their consumption pattern (<100 g/week) and maybe prefer wine. Reducing alcohol consumption is an important part of the holistic or integrated care management of AF [61]. Adherence to the latter approach is associated with improved clinical outcomes [62] and hence, recommended in guidelines [63].

## References

[1] Martignani C, Massaro G, Biffi M, Ziacchi M, Diemberger I (2020). Atrial fibrillation: an arrhythmia that makes healthcare systems tremble. *Journal of Medical Economics*.

[2] Roth GA, Mensah GA, Johnson CO, Addolorato G, Ammirati E, Baddour LM (2020). Global Burden of Cardiovascular Diseases and Risk Factors, 1990–2019: Update From the GBD 2019 Study. *Journal of the American College of Cardiology*.

[3] Lippi G, Sanchis-Gomar F, Cervellin G (2021). Global epidemiology of atrial fibrillation: An increasing epidemic and public health challenge. *International Journal of Stroke*.

[4] Kornej J, Börschel CS, Benjamin EJ, Schnabel RB (2020). Epidemiology of Atrial Fibrillation in the 21st Century: Novel Methods and New Insights. *Circulation Research*.

[5] Burdett P, Lip GYH (2022). Atrial fibrillation in the UK: predicting costs of an emerging epidemic recognizing and forecasting the cost drivers of atrial fibrillation-related costs. *European Heart Journal. Quality of Care & Clinical Outcomes*.

[6] Nalliah CJ, Sanders P, Kalman JM (2018). The Impact of Diet and Lifestyle on Atrial Fibrillation. *Current Cardiology Reports*.

[7] Manthey J, Shield KD, Rylett M, Hasan OSM, Probst C, Rehm J (2019). Global alcohol exposure between 1990 and 2017 and forecasts until 2030: a modelling study. *Lancet*.

[8] GBD 2016 Alcohol Collaborators (2018). Alcohol use and burden for 195 countries and territories, 1990-2016: a systematic analysis for the Global Burden of Disease Study 2016. *Lancet*.

[9] World Health Organization (2022). Alcohol. https://www.who.int/news-room/fact-sheets/detail/alcohol.

[10] Devos-Comby L, Lange JE (2008). “My drink is larger than yours”? A literature review of self-defined drink sizes and standard drinks. *Current Drug Abuse Reviews*.

[11] Surma S, Więcek A (2022). Alcohol and health. Is regular drinking of small doses of alcohol really good for your health?. *Archives of Medical Sciences. Atherosclerotic Diseases*.

[12] Visseren FLJ, Mach F, Smulders YM, Carballo D, Koskinas KC, Bäck M (2021). 2021 ESC Guidelines on cardiovascular disease prevention in clinical practice. *European Heart Journal*.

[13] Snetselaar LG, de Jesus JM, DeSilva DM, Stoody EE (2021). Dietary Guidelines for Americans, 2020-2025: Understanding the Scientific Process, Guidelines, and Key Recommendations. *Nutr Today*.

[14] Voskoboinik A, Prabhu S, Ling LH, Kalman JM, Kistler PM (2016). Alcohol and Atrial Fibrillation: A Sobering Review. *Journal of the American College of Cardiology*.

[15] Voskoboinik A, Marcus GM (2020). The Impact of Alcohol Intake on Atrial Fibrillation. *Current Cardiology Reports*.

[16] Linz B, Hertel JN, Jespersen T, Linz D (2022). Mechanisms and Therapeutic Opportunities in Atrial Fibrillation in Relationship to Alcohol Use and Abuse. *The Canadian Journal of Cardiology*.

[17] Tu SJ, Gallagher C, Elliott AD, Linz D, Pitman BM, Hendriks JML (2021). Risk Thresholds for Total and Beverage-Specific Alcohol Consumption and Incident Atrial Fibrillation. *JACC: Clinical Electrophysiology*.

[18] Ravens U (2003). Mechano-electric feedback and arrhythmias. *Progress in Biophysics and Molecular Biology*.

[19] Teng J, Loukin S, Kung C (2014). Mechanosensitive Ion Channels in Cardiovascular Physiology. *Experimental and Clinical Cardiology*.

[20] Sutanto H, Cluitmans MJM, Dobrev D, Volders PGA, Bébarová M, Heijman J (2020). Acute effects of alcohol on cardiac electrophysiology and arrhythmogenesis: Insights from multiscale in silico analyses. *Journal of Molecular and Cellular Cardiology*.

[21] Tonelo D, Providência R, Gonçalves L (2013). Holiday heart syndrome revisited after 34 years. *Arquivos Brasileiros De Cardiologia*.

[22] Yan J, Thomson JK, Zhao W, Gao X, Huang F, Chen B (2018). Role of Stress Kinase JNK in Binge Alcohol-Evoked Atrial Arrhythmia. *Journal of the American College of Cardiology*.

[23] Yan J, Bare DJ, DeSantiago J, Zhao W, Mei Y, Chen Z (2021). JNK2, a Newly-Identified SERCA2 Enhancer, Augments an Arrhythmic [Ca^2+^]*_SR* Leak-Load Relationship. *Circulation Research*.

[24] Voskoboinik A, Wong G, Lee G, Nalliah C, Hawson J, Prabhu S (2019). Moderate alcohol consumption is associated with atrial electrical and structural changes: Insights from high-density left atrial electroanatomic mapping. *Heart Rhythm*.

[25] Marcus GM, Dukes JW, Vittinghoff E, Nah G, Badhwar N, Moss JD (2021). A Randomized, Double-Blind, Placebo-Controlled Trial of Intravenous Alcohol to Assess Changes in Atrial Electrophysiology. *JACC: Clinical Electrophysiology*.

[26] Sha R, Rong B, Zhang K, Chen T, Wang J, Han W (2022). The role of alcohol consumption on echocardiographic and electrophysiologic changes in atrial fibrillation. *Echocardiography*.

[27] Aune D, Schlesinger S, Norat T, Riboli E (2018). Tobacco smoking and the risk of atrial fibrillation: A systematic review and meta-analysis of prospective studies. *European Journal of Preventive Cardiology*.

[28] Kaul R, Kaul R, Kaul P, Maksymiuk V, Frishman WH, Aronow WS (2022). Alcohol and Atrial Fibrillation: A Pathophysiologic Perspective. *Cardiology in Review*.

[29] Weiskirchen S, Weiskirchen R (2016). Resveratrol: How Much Wine Do You Have to Drink to Stay Healthy?. *Advances in Nutrition*.

[30] Chong E, Chang SL, Hsiao YW, Singhal R, Liu SH, Leha T (2015). Resveratrol, a red wine antioxidant, reduces atrial fibrillation susceptibility in the failing heart by PI3K/AKT/eNOS signaling pathway activation. *Heart Rhythm*.

[31] Surma S, Romańczyk M, Filipiak KJ, Lip GYH (2022). Coffee and cardiac arrhythmias: Up-date review of the literature and clinical studies. *Cardiology Journal*.

[32] Samokhvalov AV, Irving HM, Rehm J (2010). Alcohol consumption as a risk factor for atrial fibrillation: a systematic review and meta-analysis. *European Journal of Cardiovascular Prevention and Rehabilitation*.

[33] Kodama S, Saito K, Tanaka S, Horikawa C, Saito A, Heianza Y (2011). Alcohol consumption and risk of atrial fibrillation: a meta-analysis. *Journal of the American College of Cardiology*.

[34] Gallagher C, Hendriks JML, Elliott AD, Wong CX, Rangnekar G, Middeldorp ME (2017). Alcohol and incident atrial fibrillation - A systematic review and meta-analysis. *International Journal of Cardiology*.

[35] Larsson SC, Drca N, Wolk A (2014). Alcohol consumption and risk of atrial fibrillation: a prospective study and dose-response meta-analysis. *Journal of the American College of Cardiology*.

[36] Yang L, Chen H, Shu T, Pan M, Huang W (2022). Risk of incident atrial fibrillation with low-to-moderate alcohol consumption is associated with gender, region, alcohol category: a systematic review and meta-analysis. *Europace*.

[37] Zhang HZ, Shao B, Wang QY, Wang YH, Cao ZZ, Chen LL (2022). Alcohol Consumption and Risk of Atrial Fibrillation: A Dose-Response Meta-Analysis of Prospective Studies. *Frontiers in Cardiovascular Medicine*.

[38] Giannopoulos G, Anagnostopoulos I, Kousta M, Vergopoulos S, Deftereos S, Vassilikos V (2022). Alcohol Consumption and the Risk of Incident Atrial Fibrillation: A Meta-Analysis. *Diagnostics*.

[39] Jiang H, Mei X, Jiang Y, Yao J, Shen J, Chen T (2022). Alcohol consumption and atrial fibrillation risk: An updated dose-response meta-analysis of over 10 million participants. *Frontiers in Cardiovascular Medicine*.

[40] Ariansen I, Reims HM, Gjesdal K, Olsen MH, Ibsen H, Devereux RB (2012). Impact of alcohol habits and smoking on the risk of new-onset atrial fibrillation in hypertensive patients with ECG left ventricular hypertrophy: the LIFE study. *Blood Pressure*.

[41] Cha MJ, Oh GC, Lee H, Park HE, Choi SY, Oh S (2020). Alcohol consumption and risk of atrial fibrillation in asymptomatic healthy adults. *Heart Rhythm*.

[42] Csengeri D, Sprünker NA, Di Castelnuovo A, Niiranen T, Vishram-Nielsen JK, Costanzo S (2021). Alcohol consumption, cardiac biomarkers, and risk of atrial fibrillation and adverse outcomes. *European Heart Journal*.

[43] Han M, Lee SR, Choi EK, Choi J, Chung J, Park SH (2022). Habitual Alcohol Intake and Risk of Atrial Fibrillation in Young Adults in Korea. *JAMA Network Open*.

[44] Marcus GM, Modrow MF, Schmid CH, Sigona K, Nah G, Yang J (2022). Individualized Studies of Triggers of Paroxysmal Atrial Fibrillation: The I-STOP-AFib Randomized Clinical Trial. *JAMA Cardiology*.

[45] Marcus GM, Vittinghoff E, Whitman IR, Joyce S, Yang V, Nah G (2021). Acute Consumption of Alcohol and Discrete Atrial Fibrillation Events. *Annals of Internal Medicine*.

[46] Aung S, Nah G, Vittinghoff E, Groh CA, Fang CD, Marcus GM (2022). Population-level analyses of alcohol consumption as a predictor of acute atrial fibrillation episodes. *Nature Cardiovascular Research*.

[47] Frederiksen TC, Christiansen MK, Benjamin EJ, Overvad K, Olsen A, Dahm CC (2022). Five-year Changes in Alcohol Intake and Risk of Atrial Fibrillation: A Danish Cohort Study. *European Journal of Preventive Cardiology*.

[48] Biddinger KJ, Emdin CA, Haas ME, Wang M, Hindy G, Ellinor PT (2022). Association of Habitual Alcohol Intake With Risk of Cardiovascular Disease. *JAMA Network Open*.

[49] Surma S, Zembala MO, Filipiak KJ (2022). From alcohol to atrial fibrillation: much ado about… Choroby Serca i Naczyń.

[50] Caballería J (2003). Current concepts in alcohol metabolism. *Annals of Hepatology*.

[51] Tolstrup JS, Wium-Andersen MK, Ørsted DD, Nordestgaard BG (2016). Alcohol consumption and risk of atrial fibrillation: Observational and genetic estimates of association. *European Journal of Preventive Cardiology*.

[52] Yang JH, Jeong JA, Kweon SS, Lee YH, Choi SW, Ryu SY (2022). Causal Association Between Alcohol Consumption and Atrial Fibrillation: A Mendelian Randomization Study. *Korean Circulation Journal*.

[53] Yamashita T, Arima Y, Hoshiyama T, Tabata N, Sueta D, Kawahara Y (2022). Effect of the *ALDH2* Variant on the Prevalence of Atrial Fibrillation in Habitual Drinkers. *JACC: Asia*.

[54] Dixit S, Alonso A, Vittinghoff E, Soliman EZ, Chen LY, Marcus GM (2017). Past alcohol consumption and incident atrial fibrillation: The Atherosclerosis Risk in Communities (ARIC) Study. *PLoS ONE*.

[55] Choi YJ, Han KD, Choi EK, Jung JH, Lee SR, Oh S (2021). Alcohol Abstinence and the Risk of Atrial Fibrillation in Patients With Newly Diagnosed Type 2 Diabetes Mellitus: A Nationwide Population-Based Study. *Diabetes Care*.

[56] Grindal AW, Sparrow R, McIntyre WF, Conen D, Healey JS, Wong J (2022). Alcohol consumption and arrhythmia recurrence after atrial fibrillation ablation: a systematic review and meta-analysis. *Circulation*.

[57] Voskoboinik A, Kalman JM, De Silva A, Nicholls T, Costello B, Nanayakkara S (2020). Alcohol Abstinence in Drinkers with Atrial Fibrillation. *The New England Journal of Medicine*.

[58] Takahashi Y, Nitta J, Kobori A, Sakamoto Y, Nagata Y, Tanimoto K (2021). Alcohol Consumption Reduction and Clinical Outcomes of Catheter Ablation for Atrial Fibrillation. *Circulation. Arrhythmia and Electrophysiology*.

[59] Sagawa Y, Nagata Y, Miwa N, Yamaguchi T, Watanabe K, Kaneko M (2022). Alcohol Consumption Is Associated With Postablation Recurrence but Not Changes in Atrial Substrate in Patients With Atrial Fibrillation: Insight from a High-Density Mapping Study. *Journal of the American Heart Association*.

[60] Conen D (2015). Alcohol consumption and incident cardiovascular disease: not just one unifying hypothesis. *European Heart Journal*.

[61] Lip GYH (2017). The ABC pathway: an integrated approach to improve AF management. *Nature Reviews. Cardiology*.

[62] Romiti GF, Pastori D, Rivera-Caravaca JM, Ding WY, Gue YX, Menichelli D (2022). Adherence to the ‘Atrial Fibrillation Better Care’ Pathway in Patients with Atrial Fibrillation: Impact on Clinical Outcomes-A Systematic Review and Meta-Analysis of 285,000 Patients. *Thrombosis and Haemostasis*.

[63] Chao TF, Joung B, Takahashi Y, Lim TW, Choi EK, Chan YH (2022). 2021 Focused Update Consensus Guidelines of the Asia Pacific Heart Rhythm Society on Stroke Prevention in Atrial Fibrillation: Executive Summary. *Thrombosis and Haemostasis*.

